# Supplementary material for: microRNA Expression Dynamics in Culicoides sonorensis Biting Midges Following Blood-Feeding
Source: Insects. 2023 Jul 6;14(7):611. doi: 10.3390/insects14070611 (PMC10380374; doi:10.3390/insects14070611)
Supplement: Supplementary file 1 [file insects-14-00611-s001.zip › Figure S3.pdf]

>cso-bantam
CGGUUUUCAUAUUCGAUCUUACUUGUUUAUACGUAAAAGUGAGAUCAUUAUGAAAGCUAAU
>cso-let-7
UGAGGUAGUAGGUUGUAUAGUAUUGAUUCAAUAUUUACUAUUCAAUCUCCUAGCUUUGU
>cso-miR-1
CCAUGCUUCCUUGCAUUCAAUAGUAAUGAUAAGCAUAUGGAAUGUAAAGAAGUAUGGAG
>cso-miR-10
ACCCUGUAGAUCCGAAUUUGUUUUUAAUUUGAUGUAACAAAUUCGGUUCUAGAGAGGUUU
>cso-miR-100
AACCCGUAGUUCCGAACUUGUGGUUUUAAAGUAAGCAAGUUCGAUAUUAUGGGUAUC
>cso-miR-1000
AUAUUGUCCUGUCACAGCAGUACAAUUAAUAUUUAGGUUACUGUUGUUUCGGGACAUUUACA
>cso-miR-1174
UUUUACAAUUCGUGGGUGUGUUAGAGCUAGACAUUCAAAAUUUCUUUUAAUUUACAU-
UUUAUUUAAAGGAUUUGUAAUCAAUAAUUGUCAGAUCUAAAUAAUACCCACUCAUUGUAAGAAG
>cso-miR-1175
GAGUUCAAUGUACUAAGUGGAGUAGUGGUCUCAUCGCUUCAUUUUAAAUACAAGUGAGAU-
UCAACUUCUCCGACUUAUUACAUGAAAACUGU
>cso-miR-12
UGAGUAUUUCAUCAGGUACUGGUUAAGUUAAAUUUCAAUAUCACCAGUACUUAUGUGAUGCUCGUU
>cso-miR-124
GGUGUUCACUGUAGGCCAAUAUGUUCAUUAGAAACCAUAAGGCACGCGGUGAAUGCCAA
>cso-miR-125
UCCCUGAGACCCUAACUUGUGACUUUUUUAUGGUUCACAAGUUUUGAUCUCUGGUAU
>cso-miR-13
UCGUAAAAAUGGUUGUGCAAUGUUGUAUUUAAUCAAAAGCGUAUCACAGCCAUUUUGACGAGUU
>cso-miR-133
AGCUGGCUGAUUUCGGGUCAAAUCUCUAUUUAAAUGUAUUUGGUCCCCUUCAACCAGCUGU
>cso-miR-137
ACGCGUAUUCUUGGGUUAUUAACACACUGUUUAUGUUGUUAUUGCUUGAGAAUACACGUAG
>cso-miR-14
GGGAGUGAGAUAGAGGCUUUGGCUUUAACUUUGAAUUUUCAGUCAGUCUUUUUCUCUCUCCUAU
>cso-miR-184
AUUCGUACCCUUAUCACUCUUACGCCCCGUGUAUUUAAGAAACUACUGGACGGAGAACUGAU-
AAGGGCUCGUGUCAC
>cso-miR-190
AGAUAUGUUUGAUAUUCUUGGUUGUUAUUUUUCACUAACCGACCAGGAAUCAAACAUAUUAUU
>cso-miR-210
CUGCUGGCCACUUCACAAGAAGUUUAUUGACCUUAUUCUUGUGCGUGUGACAGCGGCU
>cso-miR-219
UGAUUGUCCAAACGCAAUUCUUGUUCAAUAUUUAAUAUUCAAGGGUUGUGCAUGGACUAUCGCU
>cso-miR-252
CUAAGUACUAGUGCCGCAGGAGAUAUCUGAACUCCUGCUGCCCAAGUGCUUAUCG
>cso-miR-263a
AAUGGCACUGGAAGAAUUCACGGGGUUUUUAUUGAUAUUCCCCGUGUUCUCUUAGUGGCAUCAC
>cso-miR-263b
CUUGGCACUGGGAGAAUUCACAGUAGUAUCAAAAAGUUCAAUGGUCUGUGGAUCUUUUGGUGCCAUCGU
>cso-miR-275
CGCGCUAAGCAGAGACCAAGACUGUAAAUGAUUAAAUUCAAGUCAGGUACCUGAAGUAGCGCGCG
>cso-miR-276
AGCGAGGUAUAGAGUUCCUACGUUUUAUAUGAAAUUUGUAGGAACUUCAUACCGUGCUCU
>cso-miR-277

GCGUGUCAGGAGUGCAUUUACAUUGAAAUUUUGAAUAUUUGUAAAUGCACUAUCUGGUACGACA
>cso-miR-278
CCGGACAAUAGUCCUCACAGACCGUCAUUUAAUACAGGUCGGUGGGACUUUCGUCCGUUU
>cso-miR-279a
GGUGUGGUGAAGGUCUAGUACAUGUUCUAUUUAAAAAAAAAUGUAUAUUUUUUAACUCAU-
GACUAGAUUUUCACUCAUCU
>cso-miR-279b
GAUGGGUGUAUGUCUCGUGCAUAGUCAAAAUUUAAUAUUACCAACAUAUGACUAGAUUUACACUCAUCCA
>cso-miR-2796
AGGGGUUUCUUUCGGCCUCCAGCUGUUUUUAAAUGAGACUGUAGGCCGGCGGAAACUACUUGCU
>cso-miR-281
AAGAGAGCUAUCCGUCGACAGUAGGGAUUUAAAAACACUGUCAUGGAAUUGCUCUCUUU
>cso-miR-283
CAAUAUCAGCUGGUAAUUCUGGGCUAAUAUUUCAUUCAAGUUCCCGGAAUUUCAACUGAUAUUCCA
>cso-miR-285
AUUCACAACAACUGAAGUCGAUAGGUGAAUAGAUUGCAUUAGAAAUUUCUUCUAGCACCAU-
UCGAAAUCAGUUCUAGUGAAU
>cso-miR-286a
AGAGCGAUUGUCGUCAUAGUCGCUUGAUAUUUUAUUUAACAGUGACUAGACCGAACACUCGCGUCCU
>cso-miR-2942
UAUGGGUGUGUGGUCUCGUCAACCCAGUCACUGAUAGGUCAGGUAUUCGAGACCUCAUACCUCUAG
>cso-miR-2944a
AAAGGAACUUCUGCUGUGAUUCCACAUGAAAAAAAUGAAUCACAGUAGUUGUACUUUAA
>cso-miR-2944b
AAUUCACAUGUUUGCGCUGUUAGAAGGAACUAAGGUUUGAUACGUGCCAAUUUUUCAAU-
AAUCGUAUCACAGCCUAGUUACCUUAUACCAUUGCAUUCUUUUUGGUCAU
>cso-miR-2946
CCCAUGUUAAUUCUGGACUGCAGUCUCAUUUCAAAAACUUGUAGUACGGAAUAGAUAUGGGGA
>cso-miR-2a
UCAAAGUGGUUGUGAAAUGUUUUAAUAAUAUUUCAUAUCACAGCCAGCUUUGAAGA
>cso-miR-2b
CUCACAAAGUGACUGUGAAAUGUUUAUUUUUUAUUUCAUAUCACAGCCAGCUUUGAUGAGCU
>cso-miR-2c
CCAUUAGACUUGGCUGUCUUAGUAAAACUGUUAAAAAAUCUAUCACAGCCAGCUUUGAUGAGCA
>cso-miR-305
AUUGUACUUCAUCAGGUGCUCUGGUGUCAUUGAAAAGCCCGGCACAUGUUGGAGUACACACA
>cso-miR-306
UCAGGUACUGAGUGACUCUCAGUAGUAAUGAAAUUUUACUUUAAUUUUCAUACCUUU-
GACAUAUUCUGAGAGUGAGUCUGUACCUGUUG
>cso-miR-307
ACUCACUCAACCUGGGUGUGAUGCUUAUUUGCGAUUCAUCACAACCUCCUUGAGUGAGC
>cso-miR-308
CGCAGUAUUCUCCAGUGACUUUGUUUUACUUAGAAUUUCAAAUCACAGGAGUAUACUGUGAG
>cso-miR-309
CGACAAACCAUGUUCAGUUGGUGUUUCAAUUCAAAUGUCAUCACUGGGCAAAGUUUGUCGCA
>cso-miR-315
UUUUGAUUGUUGCUCAGAAAGCCUCGAAUUUGAAAAUUGACUUUCGAGCAAUAAUUAAAUCC
>cso-miR-316
CAAAAUAUCGAUUGACUUGUCUUUUUCCGCUUACUGCCGAUGAUUUUAAAGUAAAGCGACAG-
CAAACAGGAAAAGACGCCUUAAUCGAUAUUUUG
>cso-miR-33
AUGCAUUGUAGUUGCAUUUGCGUCUUUUUUAAAUAGGCAAGUACGUCUGCAAUGCAAUU

```
>cso-miR-34
UGGCAGUGUGGUUAGCUGGUUGUGUGUUACUUUUUUUAAGAUCACAACGGCUAUCGGCACUGCUCUC
>cso-miR-7
UGGAAGACUAGUGAUUUUGUUGUUUAAUAUAAAAAAAGUAACAAAAAAUCUCUUGUCUUUCUAC
>cso-miR-71
CGUACUUUCUGUUCCAUCACUCUACCUAUAGUUUAUUUAUCUGUAUCAGUUUGAAUAGA-
GUGAUGGGUACAGAAAGAACG
>cso-miR-8
CAUCUUACCGGGCAGCAUUAGAUUGACUUUAAAUUUUCUAAUACUGUCAGGUAAAGAUGUC
>cso-miR-87
ACAUCUAAAAAUUUGCUAUUUUACCUGUGCAUUAGACGAGGUGAGCAAAUUUUCAGGUGUGU
>cso-miR-927
UUUAGAAUUCCUACGCUUUACCUUUUUAAUUGAAUGGCAAAGCGUUUGGAUUCUGAAAC
>cso-miR-929
AAAUUGACUCAAGUAGGGAGUCUUGAUAUGAUUGACUCCCUAACUAGAGUCAGAUUGAU
>cso-miR-932
UCAAUUCCGUAGUGCAUUGCAGUGUGGUCAUUAAUCAAAGCUGCAAGCACAGCGGGAGUGAGG
>cso-miR-956
UCCUUAUCGCGUUUGUAAUGGUCUCGUUGGCUAAGCUAUUCAAUUUUACAUCAAAUUGCAU-
UGAAAUGCAUUUGUUGUCACUGAUUUAGUUUCGAGACCACUGCAAACCUAU
>cso-miR-957
GUUAGUUUUGUACGGGUUUUGGUGUAUUUGGUAUUCAAACCGAAUUGAAACCGUCCAAAACUGAGGC
>cso-miR-965
GGGAAAUACUGUACGUUUUAUGUGAACUUAAUUAAAAAUCAUAAGCGUAUAGCUUUUCCCCU
>cso-miR-970
AGUCGGCGUCUGUCUUAUUGGUAGAUAUAUUGAAACCUAUCAUAAGACACACGCGGCUAU
>cso-miR-971
AAAUUGUAAGUGUAGCAACAGGCGUUUUUUGUACUGCUUGGUGUUAUAUCUUACAGUGAG
>cso-miR-981
CGGGUUUCGCGAAAAACGAGCUGUUAUAAAUACUUGAUAUAAGUUCGUUGUCGACGAAACCUGCA
>cso-miR-988
GAGUUGUUUGUGGCAAUGAGAUUCGAAUAUGAAAUUAUCCCCUUGUUACAAACCUCACGC
>cso-miR-993
UACCCUGUAGUUUCCGGGCUUUUGUAAUUUAAUUAUCAGAAGCUCGACUCUACAGGUAUCU
>cso-miR-999
ACAUGGUCGUACAGGGAUCGUUGUGUGAUUAAAACAAAUGUUAACUGUAAGACUGUGUCU
>cso-miR-9a
UCUUUGGUUAUCUAGCUGUAUGAGUGUAAUUAUCGUCAUAAAGCUAGCUUACCGAAGUUA
>cso-miR-9b
UCUUUGGUGAUUUUAGCUGUAUGCUAUAUCAAAUGAAUCGCCAUGCAGCUUUAUCACCAAAUACG
>cso-miR-X1
CAAUCUCAAAACUGUAACUGUGGGAUAAAUUUAUGCAUUGAAAAGACCCACGUUACAGGAUGGGGUUACU
>cso-miR-X2
UGGCGACUCAUGAAUGAAUAGAUUUAAUUAGUUUUCUGUUCAUUCAUGAGUCGCCAUU
>cso-miR-X3
ACAUGCUACUCAUGUCACUUCGUAAAUUUGUGGAACUCGAUGUGAUGUGAUUAGUAGUGUGUAU
```

Figure S3. Precursor sequences of *Culicoides sonorensis* miRNAs. Sequences are in FASTA format.
