# Supplementary material for: microRNA Expression Dynamics in Culicoides sonorensis Biting Midges Following Blood-Feeding
Source: Insects. 2023 Jul 6;14(7):611. doi: 10.3390/insects14070611 (PMC10380374; doi:10.3390/insects14070611)
Supplement: Supplementary file 1 [file insects-14-00611-s001.zip › Figure S4.pdf]

>cso-bantam-3p
UGAGAUCAUUAUGAAAGCUAAU
>cso-let-7-5p
UGAGGUAGUAGGUUGUAUAGU
>cso-miR-1-3p
UGGAAUGUAAAGAAGUAUGGAG
>cso-miR-10-5p
ACCCUGUAGAUCCGAAUUUGUU
>cso-miR-100-5p
AACCCGUAGUUCCGAACUUGUG
>cso-miR-1000-5p
AUAUUGUCCUGUCACAGCAGU
>cso-miR-1174-3p
UCAGAUCUAAAUAAUACCCACUCA
>cso-miR-1175 -5p
AAGUGGAGUAGUGGUCUCAUCGCU
>cso-miR-12-5p
UGAGUAUUUCAUCAGGUACU
>cso-miR-124-3p
UAAGGCACGCGGUGAAUGCCAA
>cso-miR-125-5p
UCCCUGAGACCCUAACUUGUGA
>cso-miR-13-3p
UAUCACAGCCAUUUUGACGAGUU
>cso-miR-133-3p
UUGGUCCCCUUCAACCAGCUGU
>cso-miR-137-3p
UAUUGCUUGAGAAUACACGUAG
>cso-miR-14-3p
UCAGUCUUUUUCUCUCUCCUAU
>cso-miR-184-3p
UGGACGGAGAACUGAUAAGGGC
>cso-miR-190-5p
AGAUAUGUUUGAUAUUCUUGGUUG
>cso-miR-210-3p
CUUGUGCGUGUGACAGCGGCU
>cso-miR-219
AGGGUUGUGCAUGGACUAUCGCU
>cso-miR-252-5p
CUAAGUACUAGUGCCGCAGGAG
>cso-miR-263a-5p
AAUGGCACUGGAAGAAUUCACGGG
>cso-miR-263b-5p
CUUGGCACUGGGAGAAUUCACAG
>cso-miR-275-3p
UCAGGUACCUGAAGUAGCGCGCG
>cso-miR-276-3p
UAGGAACUUCAUACCGUGCUCU
>cso-miR-277-3p
UAAAUGCACUAUCUGGUACGACA
>cso-miR-278-3p
UCGGUGGGACUUUCGUCCGUUU

>cso-miR-279-3p
UGACUAGAUUUUCACUCAUCU
>cso-miR-2796-3p
GUAGGCCGGCGGAAACUACUUGCU
>cso-miR-281-5p
AAGAGAGCUAUCCGUCGACAGU
>cso-miR-283-5p
CAAUAUCAGCUGGUAAUUCUGGG
>cso-miR-285-3p
UAGCACCAUUCGAAAUCAGUUC
>cso-miR-286a-3p
UGACUAGACCGAACACUCGCGUCCU
>cso-miR-2942-3p
UAUUCGAGACCUCAUACCUCUAG
>cso-miR-2944a-5p
AAAGGAACUUCUGCUGUGAUUCC
>cso-miR-2944b-3p
AAAGGAACUUCUGCUGUGAUUCC
>cso-miR-2946
UAGUACGGAAUAGAUAUGGGGA
>cso-miR-2a-3p
UAUCACAGCCAGCUUUGAAGA
>cso-miR-2b-3p
UAUCACAGCCAGCUUUGAUGAGCU
>cso-miR-2c-3p
UCACAGCCAGCUUUGAUGAGCA
>cso-miR-305-5p
AUUGUACUUCAUCAGGUGCUC
>cso-miR-306-5p
UCAGGUACUGAGUGACUCUCA
>cso-miR-307-3p
UCACAACCUCCUUGAGUGAGC
>cso-miR-308-5p
CGCAGUAUUCUCCAGUGACUUUG
>cso-miR-309
UCACUGGGCAAAGUUUGUCGCA
>cso-miR-315-5p
UUUUGAUUGUUGCUCAGAAAGCC
>cso-miR-316-5p
UGUCUUUUUCCGCUUACUGCCG
>cso-miR-33-3p
CAAGUACGUCUGCAAUGCAAUU
>cso-miR-34-5p
UGGCAGUGUGGUUAGCUGGUUG
>cso-miR-7-5p
UGGAAGACUAGUGAUUUUGUUGUU
>cso-miR-71-5p
CAUGAAAGACAAGGUAGUGAGA
>cso-miR-8-3p
UAAUACUGUCAGGUAAAGAUGUC
>cso-miR-87-3p
GUGAGCAAAUUUUCAGGUGUGU

```
>cso-miR-927-5p
UUUAGAAUUCCUACGCUUUACC
>cso-miR-929-5p
AAAUUGACUCAAGUAGGGAGU
>cso-miR-932-5p
UCAAUUCCGUAGUGCAUUGCAGU
>cso-miR-956-3p
UUUCGAGACCACUGCAAACCUAU
>cso-miR-957-3p
UGAAACCGUCCAAAACUGAGGC
>cso-miR-965-5p
GGGGAAAUACUGUACGUUUUAUG
>cso-miR-970-3p
UCAUAAGACACACGCGGCUAU
>cso-miR-971
UUGGUGUUAUAUCUUACAGUGAG
>cso-miR-981-3p
UUCGUUGUCGACGAAACCUGCA
>cso-miR-988-3p
CCCCUUGUUACAAACCUCACGC
>cso-miR-993-3p
GAAGCUCGACUCUACAGGUAUCU
>cso-miR-999-3p
UGUUAACUGUAAGACUGUGUCU
>cso-miR-9a-5p
UCUUUGGUUAUCUAGCUGUAUGA
>cso-miR-9b-5p
UCUUUGGUGAUUUUAGCUGUAU
>cso-miR-X1-5p
CAAUCUCAAAACUGUAACUGUGGGA
>cso-miR-X2-5p
UGGCGACUCAUGAAUGAAUAGA
>cso-miR-X3-3p
UGUGAUGUGAUUAGUAGUGUGUAU
```

Figure S4. Mature sequences of *Culicoides sonorensis* miRNAs. Sequences are in FASTA format and arm is denoted.
