# Supplementary figures and images for: microRNA Expression Dynamics in Culicoides sonorensis Biting Midges Following Blood-Feeding
Source: Insects. 2023 Jul 6;14(7):611. doi: 10.3390/insects14070611 (PMC10380374; doi:10.3390/insects14070611)

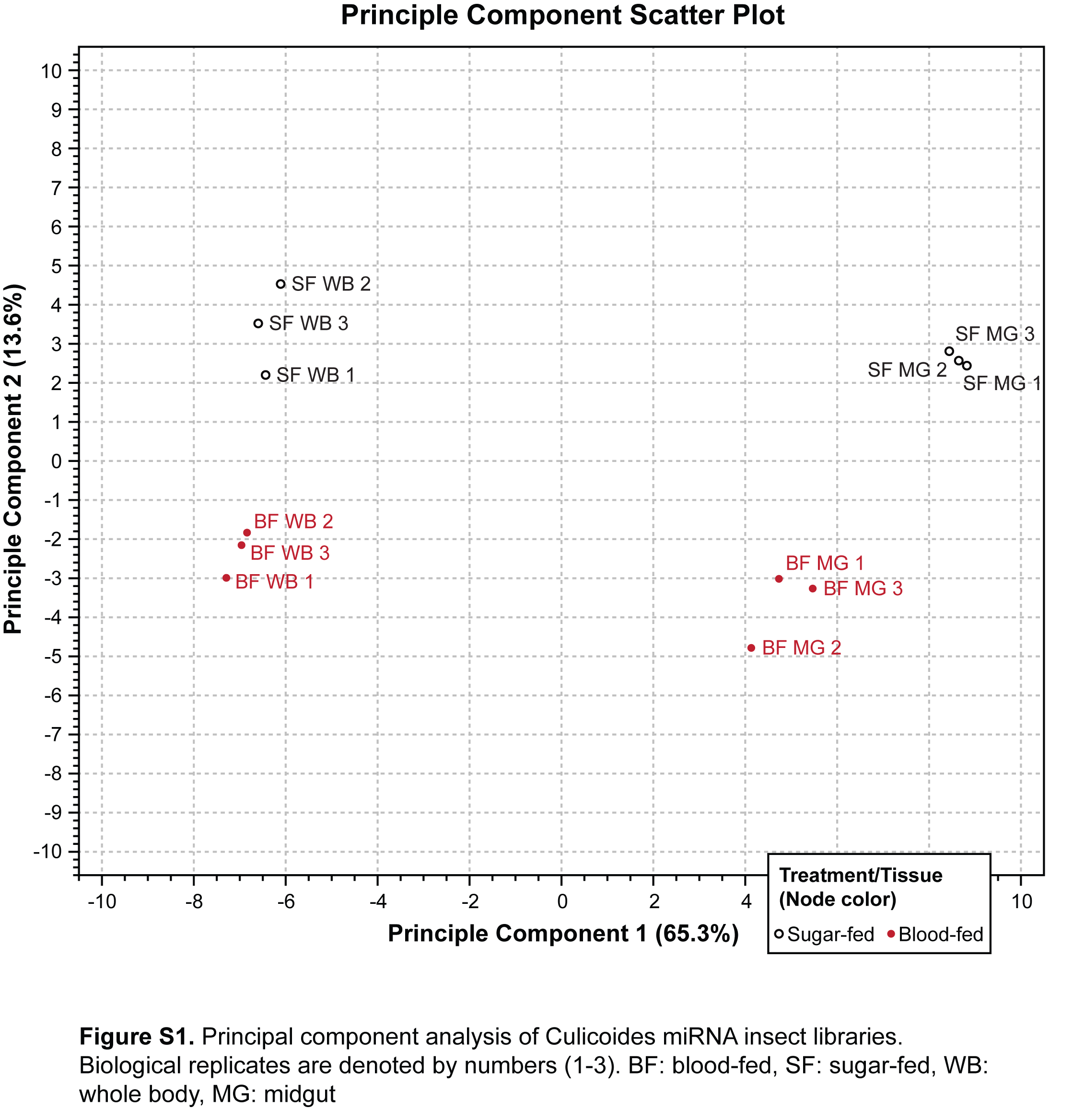

Supplement: Supplementary file 1 [file insects-14-00611-s001.zip › Figure S1.tif]

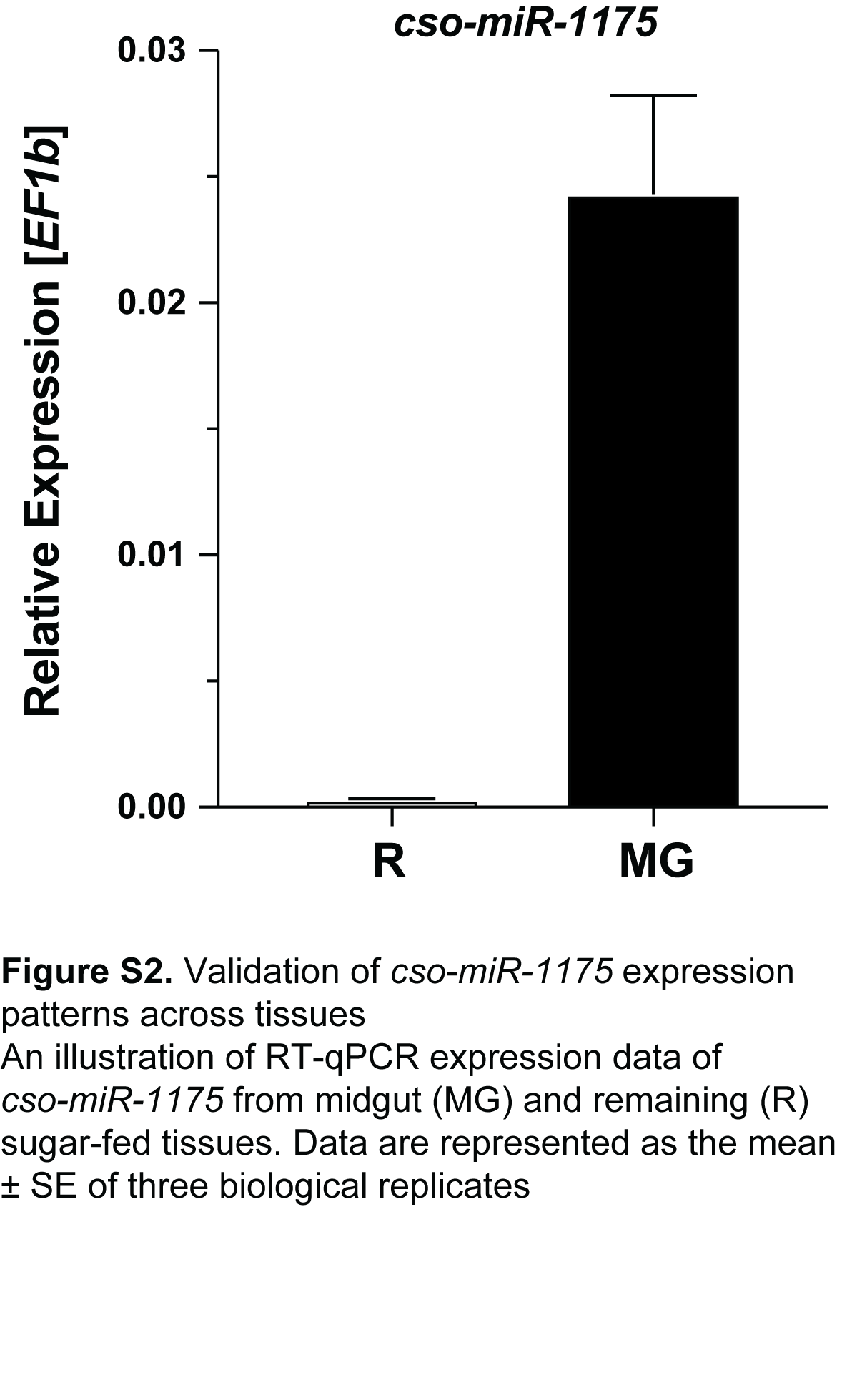

Supplement: Supplementary file 1 [file insects-14-00611-s001.zip › Figure S2.tif]
